# Bibliographical Department

**Published:** 1880-04

**Authors:** 


### BIBLIOGRAPHICAL DEPARTMENT.

"Nothing extenuate, nor set down aught in malice."

"Headaches: their Nature, Cause and Treatment." By William Henry Day, M. D., member of the Royal College of Physicians of London, Physician to the Samaritan Hospital for Women and Children. "The first requisite for success in life is to be a good animal": Herbert Spencer. Third edition, with illustrations. Philadelphia. Lindsay & Blakiston, 1880.

The foregoing is the title of a nicely gotten-up little volume of 332 pages, including index, for which we are indebted to the courtesy of the publishers. An examination of the contents leaves a very favorable impression of the author's intelligent acquaintance with the subject on which he writes. His style is perspicuous and easy of comprehension, and his conclusions logical and in harmony with the views of advanced orthodox practitioners. The book will amply repay perusal to any member of the profession.

"Yellow Fever: a Nautical Disease. Its Origin and Prevention." By John Gamgee. "As far as we know, low temperature is the only agency that can be relied on safely to destroy the infection of this disease": Dr. Carpenter. "Frost puts an end suddenly to our epidemics. Art never can do better than to imitate nature": Dr. J. C. Faget. New York: D. Appleton & Company, 549 and 550 Broadway, 1879. Pages 207, including index. Even if the author's premise could be established, his conclusion would be both illegitimate and illogical, viz: that "An empty ship from the tropical Atlantic, relieved of its crew and cargo, has been closed alongside a wharf in hot weather, and an active decomposition has set in, so that when ports and hatches are opened the volume of *fetid gases* has overwhelmed the bystanders, engendering yellow fever" (page 142). Now this would be very easy of comprehension if it were true that the *germs* of yellow fever *originate* in the manner just quoted, and the remedy would be, of course, a very simple one. But was such the *ab initio* origin of the yellow fever germ? We apprehend not, as ships probably had no existence, at least the kind now in vogue—and to which the author evidently alludes—for hundreds of centuries after the autochthones filled the hills and jungles of the western coast of Africa and suffered and died with *black vomit.* Reason, analogy and experience conspire to impress the belief on our mind that yellow fever results from the action of an *entity* or *germ* upon the human organism, and not from the inhalation of a *gaseous* compound, the result of "active decomposition" in the hold of "an empty ship" from "the tropical Atlantic" or elsewhere. We can readily understand how the yellow fever germs may be kept in a state of vitality in the hold of a ship, not only during the summer but the winter also, when the temperature has not been low enough to destroy them. But according to what we see and can learn of the action of organic matter in the production and development of *other* specific diseases, notably small-pox and measles, we are forced to conclude that when a ship in the condition described by the author has been the recognized focus of the contagion, or poison if you please, of yellow fever, the germs were introduced into it in "the tropical Atlantic" from whence it sailed, or somewhere else. The tendency "of like" is ever to reproduce itself, and it is logical, at least, to conclude that the Great and Beneficent Author of *all* law—such as we do and do not comprehend—would hardly have *decreed*, almost, if not quite *unvarying laws* for the government of *other* specific contagious diseases, which we can and do know how to appreciate and understand, and left the *origin* and development of yellow fever to blind chance! We had thought that the *spontaneous*, or even *chemical*, development of *new* organic matter so problematical, if not absolutely disproved as impossible, that anything looking in either of those directions would not likely occupy the time of the philosophical mind for a moment, and yet after admitting yellow fever to be a disease *sui generis*, if not *absolutely specific*, in the most comprehensive sense of the word, the author gravely tells us how it *originates* in the hold of a ship! Did time and space permit we would like to quote from the book some quite interesting matter that may be read profitably by any one—and who in this broad land is not?—interested in the subject of yellow fever.

After treating hundreds of cases during more than one severe epidemic of yellow fever, and making ourself somewhat familiar with the morbid anatomy and the literature of the disease, we are prepared to endorse the author's statements on page 170, viz. that "Yellow fever poison is destroyed by severe frost whenever or wherever it is not protected from it, so that *it is* exterminated by this means after epidemics, and can only be renewed by importation." "There are conditions under which *it hibernates* or lives through a mild winter, etc." "But, according to *my* enquiries, the mass of *organic molecules* floating in the air *are precipitated* and *congealed by cold* at the simple temperature of white frost, and are as *effectually* disposed of, in all probability, as if a putrid piece of flesh were boiled in a hermetically sealed bottle for six hours." Does it not seem, that while in harmony with the most generally received opinion of those who have had any considerable amount of experimental knowledge of the disease under consideration, that the above quotation is in *direct conflict* with the author's statement concerning the *gaseous origin* of yellow fever? We must regard the gases generated in a ship with closed hatches, in the circumstances he describes, as capable of producing organic particles of matter, or else his subsequent statements, last quoted, must fall to the ground! But so thoroughly have we been convinced, on theoretical ground, that we ventured to recommend to our municipal authorities, within the past two years through a leading newspaper of this city, the practical application of the freezing process to infected ships arriving in this port, for the purpose of destroying the yellow fever germs, and not for the removal of "foul gases generated in a ship with hatches closed," etc. But if the *foul gases* are the *fons et origo*, as the author seems to think, other means equally effective and far less expensive should be resorted to for the destruction of such gases, and consequent prevention of the terrible pestilence in our cities and towns. Notwithstanding the anomalous positions of the author on the origin and mode of protecting our cities against epidemics of yellow fever, there is much in the book not only calculated to interest but instruct the reader, and his complimentary remarks of Mrs. Elizabeth Thompson are both appropriate and gracefully rendered.

Note.—We have italicised several words in the quotations made from the author for reasons that must appear obvious to the reader.—Ed.

We are in the receipt of the "Transactions of the American Medical Association," Vol. 30, 1879. It is gotten up in very good style, of cloth binding, and contains 1028 pages, including index. Several of the articles are, what might have been expected from the well-known character of the authors, very good and useful productions. These will be readily recognized by the reader, and we will therefore avoid making invidious distinction, by omitting to name them. The volume contains much valuable statistical and other matter of general interest to the profession. We return thanks for our copy, probably to Dr. Atkinson, the accomplished and gentlemanly permanent Secretary ot the Association, as no name of the sender reached us.

The following journals, pamphlets, etc., have been received since our last issue, viz:

"Bulletin de l'Académie Royal de Médecine de Belgique." Année 1880. Troisième Série. Tome XIV, No. 1. Bruxelles: H. Manceaux, Imprimeur de l'Académie, Rue des Trois-Têtes, 12. (Montagne de la Cour, 1880.) This number contains much interesting matter, one article of which we hope to present to our readers in English in the next issue of this journal.

"The Southern Medical Record." A Monthly Journal of Practical Medicine. Terms, $2.00 per annum, in advance. Edited by Drs. T. S. Powell, W. T. Goldsmith and R. C. Word. Atlanta, Ga., February, 1880. Contains 40 pages of interesting matter to the profession.

"Walsh's Retrospect." A Quarterly Compendium of American Medicine and Surgery. Edited by Ralph Walsh, M.D., and Thomas E. McArdle, A.M., M.D. January, 1880. Washington, D. C.: W. H. & O. H. Morrison, publishers. We extend cordial greeting to this journal and wish it abundant success in the field of usefulness it has chosen for its labors, and if the issue before us is an earnest ot those in the future its success is assured.

"The Transactions." A bi-monthly journal of medicine and surgery, for January, comes to us from Youngstown, Ohio. Drs. Henry G. Cornwell, J. F. Wilson and M. S. Clark, editors. It is published in the interest of Mahoning County Medical Association, and is a very readable little pamphlet, at the low price of 50 cents a year, in advance.

"The Medical Tribune." A monthly journal devoted to medicine, surgery and the collateral sciences, has also been sent to us for exchange. Drs. Alexander Wilder and Robert A. Gunn. Nickles Publishing Company, 697 Broadway, N. Y. It contains, among other interesting matter, an article on Opium-Smoking in relation to the infrequency of lung and bronchial diseases, which is worthy the attention of the profession. The statements it contains would seem to point to the demoralizing and pernicious practice as prophylactic of consumption in a great degree.

"The Herald of Health." A Manual of Practical Hygiene, E. W. Gray, editor, comes to us from Bloomington, Ills. March, 1880. Terms, 50 cents a year, in advance.

"On the Relations of the Placenta to Post-partum Hemorrhage." By Walter Coles, M. D., consulting physician to St. Ann's Lying-in Asylum, St. Louis. Read before the St. Louis Medical Society, March 5th, 1880.

"Notes on the Anatomical Relations of Uterine Structures, with Surgical Remarks and Therapeutical Suggestions." By T. H. Buckler, M. D., Baltimore, Md.

"A Plea for Cold Climates in the Treatment of Pulmonary Consumption." Minnesota as a Health Resort. By Talbot Jones, M. D., of St. Paul, Minn.

"Iodoform, an important Therapeutic Agent." By Stiles Kennedy, M. D. From W. R. Warner & Co., Philadelphia.

"Twelfth Annual Report" of the New York Orthopædic Dispensary and Hospital for Children.

We are indebted to Dr. William H. Stokes, Physician in chief to Mount Hope Retreat, for the "Thirty-Seventh Annual Report" of that institution.

The April number of "The Phrenological Journal and Science of Health," published by S. R. Wells & Co., 737 Broadway, New York, has been received. It is published monthly at $2.00 per annum for single copy, and is filled with generally interesting matter, whilst treating to a certain extent on its specialty also.

"Treatment of Diphtheria." By Ira E. Oatman, M. D., of Sacramento, California.

"The New Anæsthetic, the Bromide of Ethyl." By R. J. Levis, M. D., Surgeon to the Pennsylvania Hospital and to the Jefferson Medical College Hospital.

We are kindly indebted to Dr. C. T. Stockwell, Sect., Springfield, Mass., for a copy of the "Records of the Connecticut Valley Dental

Society, for thirteen years from its organization, Nov. 10, 1863, with constitution, by-laws, code of ethics, and lists of officers and members." We are pleased to copy and fully endorse Article III. of the code of ethics of the above society, viz. "The Relative Duties of Dentists and Physicians":

"Dental surgery is a specialty in medical science. Physicians and dentists should both bear this in mind. The dentist is professionally limited to diseases of the dental organs and the mouth. With these he should be more familiar than the general practitioner is expected to be; and while he recognizes the superiority of the physician in regard to diseases of the general system, the latter is under equal obligations to respect his higher attainments in his specialty. When this principle governs, there can be no conflict or even diversity of professional interests."

TIT FOR TAT.—A medical practitioner, urgently wanting patients, and not understanding the difference between attracting and disgusting, circulates post-cards through the city addressed to any gentleman of sufficient eminence to draw his attention, on which he offers his services to cure them of fits, falling sickness, epilepsy, and all the ills too disagreeable to mention, that flesh is heir to; closing with the agreeable assurance that he will treat them in perfect confidence. Imagine his disgust on receiving from a witty lawyer this response, also spread on a post-card: "Dear Sir:—I offer you my services to defend you on your trial for murder, arson, robbery, larceny, malpractice, criminal abortion, indecent assault, body snatching, and obscene communications. I can secure, if not your acquittal, at least the mitigation of punishment, every time. N. B.—This post-card is strictly confidential."—*Albany Law Journal.*

